# Characterising cellular and molecular features of human peripheral nerve degeneration

**DOI:** 10.1186/s40478-020-00921-w

**Published:** 2020-04-17

**Authors:** Matthew B. Wilcox, Simão G Laranjeira, Tuula M. Eriksson, Kristjan R. Jessen, Rhona Mirsky, Tom J. Quick, James B. Phillips

**Affiliations:** 1 Peripheral Nerve Injury Research Unit, Royal National Orthopaedic Hospital, Stanmore, UK; 2 Department of Pharmacology, UCL School of Pharmacy, University College London, London, WC1N 1AX UK; 3 UCL Centre for Nerve Engineering, University College London, London, UK; 4 Department of Mechanical Engineering, University College London, London, UK; 5 Department of Biomaterials and Tissue Engineering, Eastman Dental Institute, University College London, London, UK; 6 Department of Cell and Developmental Biology, University College London, London, UK

**Keywords:** Peripheral nerve degeneration, Human tissue, Nerve transfer, Muscle reinnervation, Schwann cells

## Abstract

Nerve regeneration is a key biological process in those recovering from neural trauma. From animal models it is known that the regenerative capacity of the peripheral nervous system (PNS) relies heavily on the remarkable ability of Schwann cells to undergo a phenotypic shift from a myelinating phenotype to one that is supportive of neural regeneration. In rodents, a great deal is known about the molecules that control this process, such as the transcription factors c-Jun and early growth response protein 2 (EGR2/KROX20), or mark the cells and cellular changes involved, including SOX10 and P75 neurotrophin receptor (p75NTR). However, ethical and practical challenges associated with studying human nerve injury have meant that little is known about human nerve regeneration.

The present study addresses this issue, analysing 34 denervated and five healthy nerve samples from 27 patients retrieved during reconstructive nerve procedures. Using immunohistochemistry and Real-Time quantitative Polymerase Chain Reaction (RT-qPCR), the expression of SOX10, c-Jun, p75NTR and EGR2 was assessed in denervated samples and compared to healthy nerve. Nonparametric smoothing linear regression was implemented to better visualise trends in the expression of these markers across denervated samples.

It was found, first, that two major genes associated with repair Schwann cells in rodents, c-Jun and p75NTR, are also up-regulated in acutely injured human nerves, while the myelin associated transcription factor EGR2 is down-regulated, observations that encourage the view that rodent models are relevant for learning about human nerve injury. Second, as in rodents, the expression of c-Jun and p75NTR declines during long-term denervation. In rodents, diminishing c-Jun and p75NTR levels mark the general deterioration of repair cells during chronic denervation, a process thought to be a major obstacle to effective nerve repair. The down-regulation of c-Jun and p75NTR reported here provides the first molecular evidence that also in humans, repair cells deteriorate during chronic denervation.

## Introduction

Peripheral Nerve Injury (PNI) results in partial or complete loss of sensory and/or motor function and involvement of sympathetic and pain systems in the body segment involved. A study estimates that the incidence of PNI is 560,000 cases per year in the United States alone [1]. The debilitating effects of PNI are highlighted by the much greater prevalence than incidence which leads to long term disruption in the lives of patients [2].

The cellular and molecular mechanisms that underpin nerve regeneration have been investigated extensively in animal models demonstrating that the plasticity of Schwann cells and their ability to switch to a repair–supportive differentiation state after injury is one of the key reasons for strong regenerative capacity observed in the Peripheral Nervous System (PNS) [3–7].

In rodent models, it has been shown that the failure of motor recovery after chronic denervation (greater than 6 months) is associated with substantially reduced capacity of the distal nerve to support growth of axons [8–10]. After injury, progressive deterioration of denervated nerve and muscle make conditions increasingly antagonistic for regeneration, decreasing the chance of functional recovery [11]. Rodent studies have established that this can largely be attributed to adverse changes in Schwann cells and their associated basal laminae, including reduced expression of repair-supportive molecules and decreasing Schwann cell numbers [12–14]. Although this process is thought to be a major obstacle to effective nerve repair, this progressive loss of regeneration support has not been investigated in detail in humans.

Clinical reports have suggested that optimal functional recovery is dependent upon a sufficient number and quality of axons reaching their target within 1 year following injury [15–18]. After this time period, functional outcomes are poor [18–20]. This same pattern of strong initial regeneration potential followed by declining regenerative capacity during chronic denervation and poor functional outcomes from repair of proximal injuries suggests that the basic biology is likely to be comparable between rodents and humans.

Effective translation of the wealth of animal model data into a human paradigm of nerve regeneration would be of great benefit in the development of improved clinical treatments for nerve injury, but progress is limited by ethical and practical challenges associated with studying human nerve injury [21, 22]. Moreover, the intricate anatomy and diverse range of injuries make PNI a heterogeneous pathology to study.

**Fig. 1:**
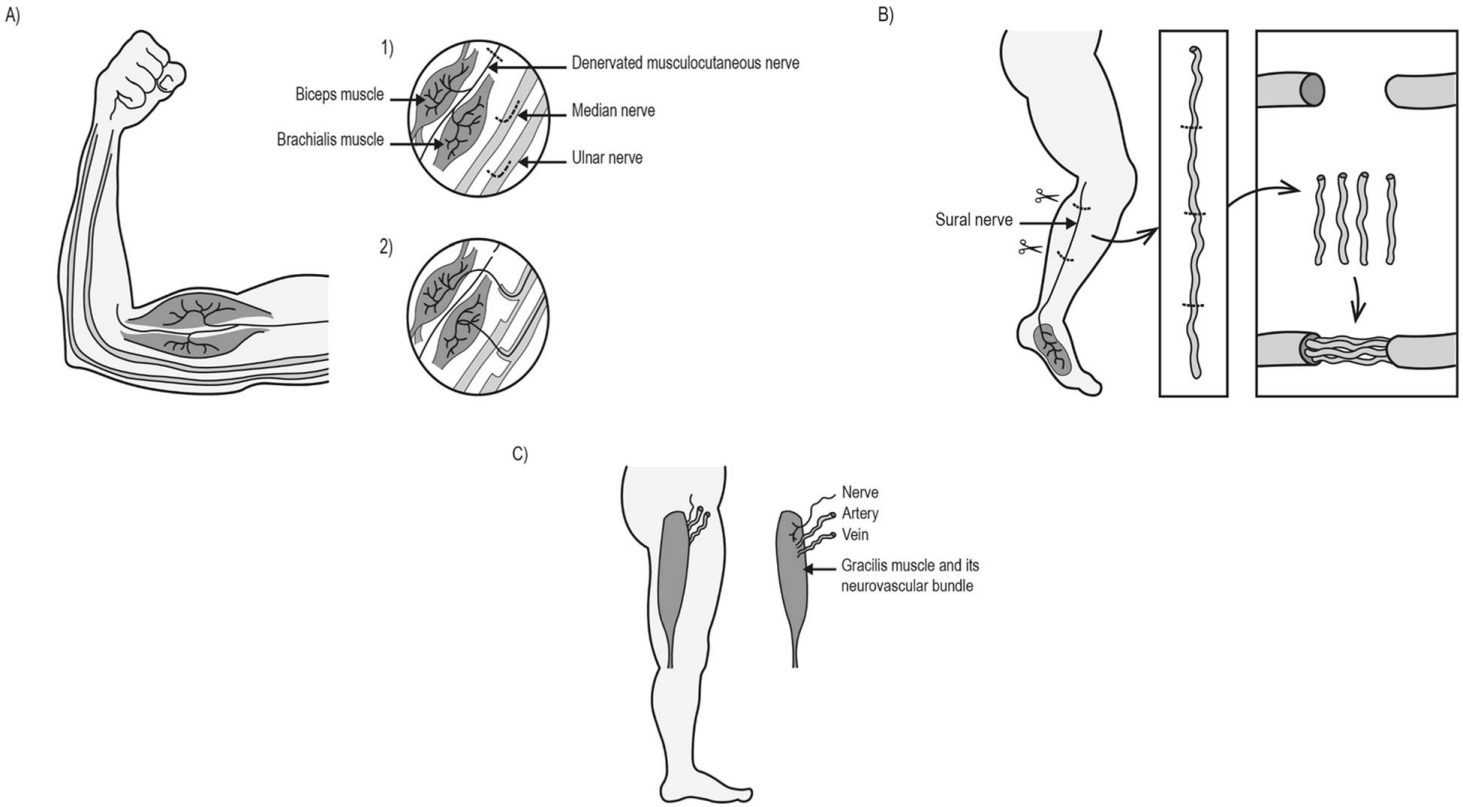
Reconstructive nerve procedures. **a** The double Oberlin’s nerve transfer is commonly deployed to restore elbow flexion. The surgeon identifies suitable donor fascicles of the ulnar and median nerve that supply wrist flexor muscles. The fascicles are divided and redirected to grow into the denervated musculocutaneous nerve to biceps and brachialis. **b** Nerve autograft is deployed in larger nerve gaps. The sural nerve is often harvested as the donor nerve and grafted to restore continuity across the damaged nerve trunk. **c** Free functional muscle transfer (FFMT) is deployed in chronic nerve injuries. This involves identifying a suitable donor muscle and its neurovascular bundle (such as the gracilis) and grafting it to the injured site of nerve damage (often to the upper limb to restore elbow flexion)

To address this challenge, this study retrieved nerve samples from patients undergoing treatment for nerve injuries at a range of times from injury from acute to chronic. Many nerve samples were retrieved from nerve transfer surgeries. This procedure is deployed by the reconstructive nerve surgeon following complex proximal nerve injuries or those where there has been a significant delay from injury to treatment. The damaged nerves were identified and characterised using intra-operative neurophysiological monitoring to record Compound Nerve Action Potentials (CNAPs). In line with current practice [23, 24], the nerve was assumed to be denervated if a CNAP and muscle twitch were absent. The surgeon identified and isolated a suitable donor fascicle and created a neurotmesis injury in order to redirect previously uninjured axons to grow into the chronically denervated stump. The Oberlin’s procedure is an example of a nerve transfer which is commonly used to reanimate elbow flexion (Fig. 1a) [17, 25, 26]. A nerve autograft is another surgical technique which can be deployed to reconstruct a nerve gap where the timing and local tissue conditions allow. For this the medial cutaneous nerve of the arm or the sural nerve are commonly utilised as the donor (Fig. 1b) [27]. In cases where significant time (greater than 1 year) has passed since the initial nerve injury, a Free Functioning Muscle Transfer (FFMT) is often the only technique to restore movement (Fig. 1c) [27]. This involves identifying a suitable donor muscle as well as its neurovascular bundle and grafting it in order to restore a function considered to be more pertinent to the quality of life of the patient. All of these surgical protocols liberate excess tissue (both healthy and denervated samples) for study in the laboratory, that would otherwise be disposed of. Across our cohort, samples from various time points during chronic denervation were harvested, allowing the time course of any phenotypic changes in denervated nerve tissue to be explored for the first time. In rodent models, Schwann cells reprogram to a transient pro-regenerative phenotype, the ‘repair Schwann cell’. Therefore, in the present study we analysed key markers linked with the transition to the repair Schwann cell phenotype, c-Jun, p75 Neurotrophin receptor (NTR) and early growth response protein 2 (EGR2/KROX20), in addition to a pan-Schwann cell maker, SOX10.

Upregulation of the transcription factor c-Jun in Schwann cells follows nerve injury and amplifies a cascade of downstream changes in expression associated with the phenotypic shift of Schwann cells from a normal myelinating or Remak phenotype to a repair phenotype [28]. Other well-characterised changes associated with Schwann cell reprogramming following injury in rodents include upregulation of p75NTR and downregulation of the transcription factor EGR2 which is associated with myelination [29–32]. SOX10 is a transcription factor constitutively expressed in Schwann cells and has a crucial role in neural crest development, glial cell development and myelin formation and maintenance [33–36]. It serves as a marker to specifically identify Schwann cells in nerve samples [34, 37, 38]**.** Expression of this panel of four markers was determined in the denervated human nerve samples and compared with healthy control human nerve tissue at the level of gene expression (Real-Time quantitative Polymerase Chain Reaction, RT-qPCR) and tissue protein presence as well as distribution was quantified using immunohistochemistry.

## Methods and materials

Informed consent was obtained according to the declaration of Helsinki [39]. Ethical approval for this project was provided by the UCL Biobank Research Committee (REC 15.15). Twenty-seven patients who underwent reconstructive nerve procedures (nerve transfer, FFMT and nerve autograft) were included. The demographics of the patients identified and included in this study are outlined in Table 1. The innervation status of all nerve samples was determined by intra-operative neurophysiology; if a CNAP and muscle twitch was absent, the nerve was judged to be denervated. Nerve samples were obtained during the course of the surgical procedure, then processed for immunohistochemistry and/or RT-qPCR analysis. Since many of the patients had suffered global plexus injuries, healthy nerve samples were only included if they were retrieved from sites external to the injury site (the affected upper limb) to ensure the sample was not damaged.

**Table 1:** Patient demographics

Case Number	Age Range (Gender)	Mechanism of Injury	Intraoperative findings	Method from Fig. 1	Reconstructive Nerve Procedure	Details of nerve liberated (denervated unless otherwise stated)	Sample used for histology or RT-qPCR?	Denervation Period (Days)
1	20–30 (M)	Motorbike accident	Rupture	A	Right spinal accessory nerve transfer suprascapular	Distal stump of suprascapular	RT-qPCR	4
2	30–40 (M)	Fall and laceration	Rupture and Laceration	B	Medical cutaneous nerve of arm autograft to ulnar	Distal stump of ulnar	RT-qPCR	8
3	30–40 (M)	Motorbike accident	Neurotmesis	A	Spinal accessory nerve transfer to suprascapular	Distal Stump of suprascapular	RT-qPCR	12
4	20–30 (F)	Trampoline accident (radius and ulnar mid-shaft fracture)	Neurotmesis	C	Sural nerve graft to ulnar	Sural (innervated) and distal stump of ulnar	RT-qPCR	40
5	30–40 (M)	Motorbike accident	C5–7 Avulsion	A	Spinal accessory nerve transfer to suprascapular transfer and Double Oberlin’s nerve transfer	Distal stump of biceps branch of musculocutaneous and distal stump of suprascapular	RT-qPCR	42
6	20–30 (M)	Motorbike accident	C5-T1 Avulsion	A	Right intercostal nerve transfer to long thoracic nerve	Distal stump of long thoracic nerve	RT-qPCR	110
7	30–40 (M)	Glass Laceration	Laceration	N/A	Resection of left common peroneal nerve	Distal stump of common peroneal nerve	RT-qPCR	116
8	20–30 (M)	Moped v Lampost	C5–8 Avulsion	A	Intercostal nerve transfer to radial nerve	Intercostal (innervated) and distal stump of radial nerve	RT-qPCR	119
9	40–50 (M)	Motorcycle v car and bus	C5/6 Avulsion	A	Spinal accessory nerve transfer to suprascapular	Distal stump of suprascapular nerve	RT-qPCR	119
10	20–30 (M)	Car v Tree	C5/6/7 Avulsion	A	Oberlin’s nerve transfer	Distal stump of biceps branch of musculocutaneous	RT-qPCR	170
11	20–30 (M)	Motorbike accident	C5/6 Avulsion	A	Oberlin’s nerve transfer	Distal stump of biceps branch of musculocutaneous	RT-qPCR	180
12	30–40 (M)	Fall and laceration	High ulnar nerve laceration	A	Anterior interosseous nerve transfer to ulnar nerve	Distal stump of ulnar nerve	RT-qPCR	182
13	50–60 (M)	Mechanical fall	C5/6 Avulsion	A	Right anterior interosseous nerve transfer to ulnar	Distal stump of ulnar nerve	RT-qPCR	270
14	50–60 (M)	Iatrogenic - left sided neck lymph node biopsy	Neurotmesis	A	Spinal accessory nerve transfer to suprascapular	Distal stump of suprascapular	RT-qPCR	375
15	40–50 (M)	Road Traffic Accident	C5-T1 Avulsion	A	Spinal accessory nerve transfer to suprascapular	Distal stump of suprascapular	RT-qPCR	478
16	40–50 (M)	Car v Lorry	Axonotmesis	A	C7 fascicle to spinal accessorynerve	Distal stump of spinal accessory	RT-qPCR and Histology	540
17	20–30 (M)	Stab wound to the neck	C5/6 Neurotmesis	A	Oberlin’s nerve transfer	Distal stump of C6	Histology	3
18	20–30 (M)	Motorbike v Car	C5/6 Avulsion	A	Oberlin’s nerve transfer	Distal stump of biceps branch of musculocutaneous	Histology	30
19	20–30 (M)	Motorbike v Truck	Axonotmesis	A	Double Oberlin’s nerve transfer	Distal stumps of biceps and brachialis branches of musculocutaneous	Histology	42
20	50–60 (F)	Mechanical Fall	C4/5/6/7 Avulsion	A	Double Oberlin’s nerve transfer	Distal stumps of biceps and brachialis branches of musculocutaneous	Histology	58
21	20–30 (M)	Motorbike v Tree	Axonotmesis	A	Oberlin’s nerve transfer	Distal stump of biceps branch of musculocutaneous	Histology	107
22	20–30 (M)	Seizure whilst driving	Axonotmesis	A	Oberlin’s nerve transfer	Distal stump of biceps branch of musculocutaneous	Histology	172
23	20–30 (M)	Road Traffic Accident	C5/6 Avulsion	C	Free functioning muscle transfer to restore elbow flexion	Distal stump of biceps branch of musculocutaneous	Histology	4745
24	30–40 (M)	Road Traffic Accident	C5-C8 Avulsion	C	Free functioning muscle transfer to restore elbow flexion	Intercostal (innervated)	RT-qPCR	6432
25	20–30 (M)	Stab wound the neck	Neurotmesis	A	Subclavian nerve transfer to spinal accessory nerve	Distal stump of spinal accessory	Histology	62
26	20–30 (M)	Motorbike v Car	Axonotmesis	A	Nerve to long head of triceps nerve transfer to axillary nerve	Distal stump of axillary nerve	Histology	294
27	30–40 (M)	Motorbike accident	C5/6 Avulsion	A	Double Oberlin’s nerve transfer	Distal stumps of biceps and brachialis branches of musculocutaneous	Histology	115

Tabulation of the patient demographic included in this study and details of nerve sample liberated

### Immunohistochemistry

#### Staining protocols

Samples liberated from reconstructive surgical nerve procedures were immediately fixed in 10% formalin and then embedded orthogonally in paraffin wax. Serial cross-sections were cut (3 μm) using a microtome and immunostaining for neurofilament, SOX10, c-Jun, p75NTR and EGR2 performed.

All staining was carried out using the Leica Bond III automated immunostaining platform, using Leica Bond Polymer Refine Detection with a 3,3′-Diaminobenzidine (DAB)/horseradish peroxidase (HRP) chromogen (Leica, DS9800), with incubations at ambient temperature unless otherwise specified. Dewax was carried out on-board using Leica Bond Dewax solution (Leica, AR9222). Washes were performed between each step using Leica Bond Wash (Leica, AR9590). DAB was enhanced using 0.5% copper sulphate following application for 10 min.

After on-board heat-induced epitope retrieval (HIER) with Leica Epitope retrieval solution 2 (Leica ER2, high pH, AR9640) for 20 min at 99 °C, primary antibodies were applied using the following dilutions (using Leica Bond Primary Antibody Diluent (Leica, AR9352)) at ambient temperature: c-Jun (rabbit monoclonal 16A8, Cell Signalling Technologies #9165, 1:500 for 30 min), EGR2 (goat polyclonal, AbCam ab63943, 1:200 for 15 min), neurofilament (NF200 mouse monoclonal N52.1.7, Leica Biosystems PA0371, applied as supplied for 15 min). For the SOX10/p75NTR co-stain, the SOX10 primary antibody was added first (rabbit monoclonal EP268, CellMarque 383R-15, 1:200 for 15 min) followed by p75NTR (rabbit polyclonal, Novus Biologicals NBP1–85769, 1:400 for 30 min). The Leica Bond Polymer Refine Detection system (Leica, DS9800) was used for post-primary treatment of all the sections. For the SOX10/p75NTR co-stain, the Leica Bond Polymer Refine Red detection system (Leica Biosystems, DS9390) was utilised in addition. The staining signal for SOX10 and p75NTR was distinguished by nucleus and cytoplasm localisation respectively.

All immunohistochemistry protocols were validated using positive controls (documented in the Supplementary Fig. 1a, b, c and d).

#### Image capture and quantification

Micrographs were captured using the Leica ATC2000. The total cell count was quantified by manually counting the total number of haematoxylin positive cells within each fascicle using ImageJ software [40]. This provided a value for the number of cells per mm^2^. Similarly, the total number of SOX10 positive cells within each fascicle was also calculated to quantify the number of Schwann cells per mm^2^.

In order to assess the presence of c-Jun, p75NTR and EGR2 immunoreactivity in Schwann cells, double-stained sections or adjacent serial sections were quantified in the same way and related to the number of Schwann cells per mm^2^. The number of neurofilament positive fibres within each fascicle was also determined to calculate the axon density (axons per mm^2^) for each nerve sample.

### RT-qPCR

#### RNA extraction protocol

The surgical environment affords a number of challenges to the isolation of RNA from nerve samples in sufficient quantities and qualities for downstream RT-qPCR assays [21, 22, 41]. In concordance with experimental findings that have characterised the effect of peri-operative variables on the quality and quantity of RNA isolated from human peripheral nerve samples [22], this study minimised the time delay between surgical liberation and cryopreservation as well as minimising the exposure of samples to surgical antiseptics wherever possible.

Each human nerve sample was placed into a 5 ml tube and snap frozen in liquid nitrogen. RNA was isolated from all nerve samples using the Qiagen RNeasy® Fibrous Tissue Mini Kit. The Cole-Palmer® LabGEN 125 tissue homgenizer with Cole-Parmer LabGEN® Rotor-Stator Generator, Fine, 75 mm × 5 mm was used to homogenise samples. The quantity of RNA was determined using a Tecan™ Infinite 200 PRO multimode reader. Quality of RNA was measured using a NanoDrop™ spectrophotometer to ascertain 260/280 ratios for each sample. Samples were also analysed using Bio-rad Experion™ RNA analysis kits to assess Ribosomal Integrity Number (RIN), obtain electropherogram data and automated agarose gel readings from samples using the Experion™ Automated Electrophoresis System. Samples that did not have 260/280 ratios between 1.8 and 2.1 were excluded.

#### RNA to cDNA synthesis

In order to convert RNA to complementary DNA (cDNA), the Qiagen whole genome reverse transcription (RT) kit was utilised. The isolated RNA in solution was thawed on ice (within 1 week of RNA isolation from the nerve sample). A minimum of 10 ng of RNA (in 1-5 μl of RNase free water) was added to a microcentrifuge tube. The resulting volume of RNA was adjusted to equate to 5 μl by adding RNase free water. The RT mix was then prepared using the T-Script Buffer and T-Script enzyme in a ratio of 4:1 respectively. A total of 5 μl of this RT mix was added to the 5 μl solution of RNA. This mix was then placed into a thermocycler (Applied Biosystems SimpliAmp™ Thermal Cycler) and incubated at 37 °C for 30 min. After this time period, the reaction was terminated by incubating the mix at 95 °C for 5 min followed by cooling to 22 °C.

The ligation mix was then prepared using Ligation Buffer, Ligation Reagent, Ligation Enzyme 1 and Ligation Enzyme 2 in a ratio of 6:2:1:1 respectively (and added in this chronology). A total of 10 μl of this mix was added to the resultant RT mix and then vortexed. This mixture was then incubated in the thermocycler at 22 °C for 2 h. The amplification mix was then prepared by mixing REPLI-g Midi Reaction Buffer and REPLI-g Midi DNA Polymerase in a ratio of 29:1 respectively. A total of 30 μl of this reaction mix was added to the ligation mix by vortexing and centrifuging briefly. This mixture was then incubated in the thermocycler at 30 °C for 8 h (high-yield reaction). After this time period, the reaction was terminated by incubating the mixture at 95 °C for 5 min. The resultant cDNA was then diluted in RNase free water in a ratio of 1/250 (2 μl of cDNA added to 500 μl of RNase free water) and stored at -20 °C until required for downstream RT-qPCR.

#### RT-qPCR reaction mix

Primers for each gene of interest (GOI) and housekeeping gene (HKG) were designed based on the sequences validated at the Harvard Primerbank [42, 43] and supplied by Sigma-Aldrich. The sequences for the forward and reverse primers are shown for each gene in Table 2. All assays were optimised such that the efficiency of the RT-qPCR reactions was between 90 and 110% in concordance with published guidelines [44, 45].

**Table 2:** Primer sequences

***Gene***	***Forward Sequence***	***Reverse Sequence***
SOX10	AGGCTGCTGAACGAAAGTGACAAG	ACTTGTAGTCCGGGTGGTCTTTCT
c-Jun	TCCAAGTGCCGAAAAAGGAAG	CGAGTTCTGAGCTTTCAAGGT
p75NTR	TGAACGACCCCAACAATGTGG	GGCTTTTGCTGATACGCTCG
EGR2	TCTTCCCAATGATCCCAGACT	TTACGGATTGTAGAGAGTGGAGT
18S (Housekeeping gene)	CGCGGTTCTATTTTGTTGGT	CGGTCCAAGAATTTCACCTC

A table to show the sequences of forward and reverse primers used in the RT-qPCR assays

All reaction components were thawed on ice then mixed and briefly centrifuged until the reagents were at the bottom of the tubes. To set up the RT-qPCR reactions a MasterMix was made up of 10 μL of PowerUp™ SYBR™ Green MasterMix (2X), 2.5 μL of each forward and reverse (100 mM solution) primer and 5 μL of DNA template diluted in RNase free water (20 μL/Well). Sufficient MasterMix was made to run assays for GOI and HKG for each sample in triplicate. MasterMix was made up as n + 1 to allow for pipetting errors. This mixture was transferred into a 96-well optical plate (Thermofisher AB-0800).

In all reaction well plates, no template negative controls (NTC) were run along with 2 control samples (sural and two intercostal nerve samples from case numbers 4, 8 and 24 respectively shown in Table 1). For the NTC reactions, 5 μl of RNase free water was added to the well instead of cDNA template. The optical well plate was sealed with a MicroAmp™ Adhesive Optical Cover and briefly centrifuged to ensure reagents were collected at the bottom of the plate. The plate was then transferred into the Applied Biosystems (Thermofisher QuantStudio™ 3 System) to run the RT-qPCR assay using the following thermocycling parameters: an initial denaturation stage to 94 °C for 2 min followed by 40 cycles of heating to 94 °C for 15 s (denaturation), 60 °C for 1 min (annealing, extension and read fluorescence).

#### Quantification

The Livak [46] method of quantification was used to determine the relative gene expression by characterising the differential between threshold cycle (C_T)_ values for the endogenous control (18S) (C_T:e_) and the calibrator (sural/intercostal nerve) sample (C_T:c_). 18S was selected as the HKG as it has been shown to be consistently expressed across different human Schwann cell phenotypes [47].

Relative Quantification (RQ) = 2^-ΔΔCT^

with

$$ {\Delta \Delta \mathrm{C}}_{\mathrm{T}}={\Delta \mathrm{C}}_{\mathrm{T}}-{\mathrm{C}}_{\mathrm{T}:\mathrm{c}} $$

and

$$ {\Delta \mathrm{C}}_{\mathrm{T}}={\mathrm{C}}_{\mathrm{T}}-{\mathrm{C}}_{\mathrm{T}:\mathrm{e}} $$

### Statistical analysis

Nonparametric smoothing linear regression was applied to immunohistochemistry and RT-qPCR data to allow improved visualisation of the trends presented in the data. More details on how this was performed is provided in the supplementary material.

## Results

Thirty four denervated and five healthy nerve samples were included in the study, from 26 males and one female. The mean patient age at the time of surgery was 34 (±6) years. The median time between injury and surgery was 116 days (ranging from 4 to 6432 days). A total of 64 nerve samples were retrieved from surgery over a 3 year period. However, 25 samples (39%) were rejected from the study due to one of the following reasons:

1. Fourteen samples yielded insufficient quantity and/or quality of RNA for RT-qPCR analysis.
2. Seven of the samples were of insufficient quantity for sectioning.
3. Four of the samples presented an inappropriate morphology for sectioning.

For immunohistochemistry, a total of two independent nerve samples with no known pathology were retrieved as a baseline (Case number 4 and 8 as described in Table 1). The size of the intercostal nerve sample from Case number 24 (Table 1) was insufficient for immunohistochemical analysis. Quantification of immunohistochemistry suggested that across the two healthy nerve samples, the density of SOX10 cells as well as expression levels of each of the phenotypic markers c-Jun, p75NTR and EGR2 was similar (1872 ± 258 mm^2^, 193 ± 19 mm^2^, 76 ± 14 mm^2^ and 1555 ± 45 mm^2^ respectively) (Supplementary Fig. 2). Moreover, axon density was similar in sural and intercostal nerve samples (34,567 ± 1107 mm^2^) (Supplementary Fig. 3).

**Fig. 2:**
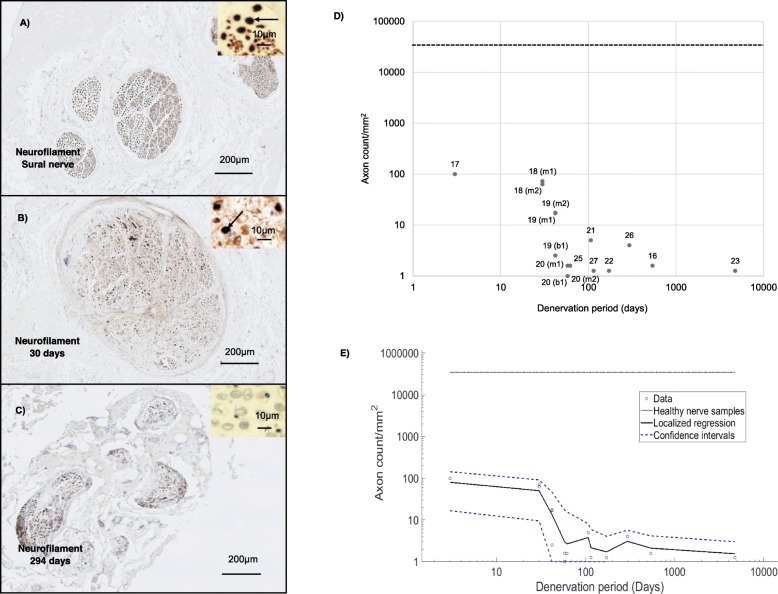
Immunohistochemical detection of neurons in healthy and denervated human nerves. **a**– **c** represent nerve cross sections stained for neurofilament (brown) with haematoxylin and eosin stain. The black arrow in the micrograph represents positive neurofilament staining. In **d** and **e** the black dotted line represents the mean number of axons detected in healthy nerve samples (case number 4 and 8). The x-axis is Log (denervation time in days). **a** Healthy sural nerve. **b** Biceps branch of the musculocutaneous nerve denervated for 30 days. **c** Axillary nerve denervated for 294 days with deteriorated morphology. **d** A scatter plot to represent Log (axon count/mm^2^) against denervation time. **e** Nonparametric smoothing linear regression of the Log (axon count/mm^2^) against denervation time. Case numbers are attached to each data point for reference to Table 1 with descriptors of whether the sample was collected proximally or distally: *m1* - Proximal part of the denervated stump of the biceps branch of musculocutaneous nerve. *m2* - Distal part of the denervated stump of the biceps branch of musculocutaneous nerve. *b1* - Proximal section of the denervated stump of the brachialis branch of musculocutaneous nerve. *s1* - Denervated stump of suprascapular nerve. *sa1* - Proximal section of the denervated stump of the spinal accessory nerve. *sa2* - Distal section of the denervated stump of the spinal accessory nerve

For RT-qPCR, a total of three independent nerve samples with no known pathology were retrieved as baseline controls (one sample of sural nerve (Case number 4) and two samples of intercostal nerve (Case number 8 and 24) as shown in Table 1). The mean relative gene expression in these control samples (where ΔC_T_ = C_T_–C_T:e_) of the SOX10, c-Jun, p75NTR and EGR2 (Supplementary Fig. 4) was relatively similar across the healthy nerve samples (13.26 ± 1.89, 10.78 ± 0.42, 12.55 ± 1.62, 8.96 ± 0.81 respectively). Immunohistochemical detection of axons in the damaged samples using neurofilament antibodies revealed that the large majority of samples contained fewer than 10 axons (Fig. 2) beyond 40 days of denervation.

**Fig. 3:**
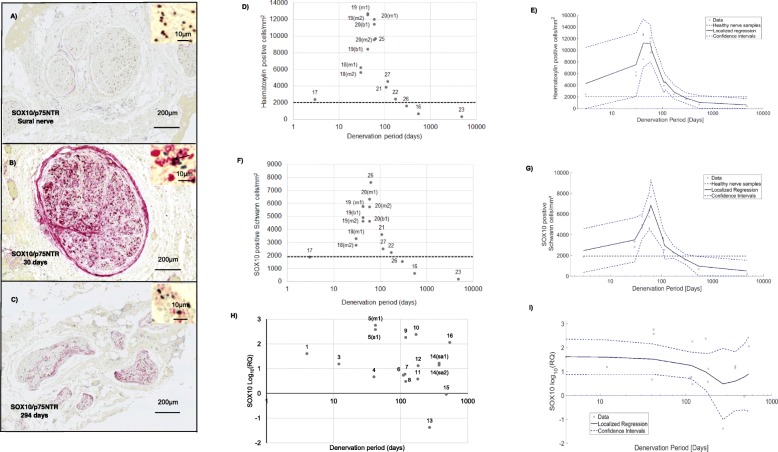
Immunohistochemical and RT-qPCR analysis of Schwann cells in healthy and denervated human nerves. **a**-**c** represent nerve cross sections immunostained for SOX10 (brown) and P75 p75NTR (red) along with haematoxylin and eosin stain. The black arrow in the micrographs indicates a SOX10/p75NTR positive Schwann cell. **d**–**i** the x-axis represents Log (denervation time in days). In **d**-**g**, the horizontal black dotted line represents the mean value obtained for the healthy nerve group. **a** Healthy sural nerve **b** Biceps branch of the musculocutaneous nerve denervated for 30 days. The brown staining represents a SOX10 positive nucleus whilst the red cytoplasmic staining represents p75NTR positive staining. **c** Axillary nerve denervated for 294 days with deteriorated morphology. **d** Scatter plot to represent the total number of haematoxylin positive cells/mm^2^ in denervated samples. **e** Nonparametric smoothing linear regression of the total number of haematoxylin positive cells/mm^2^ in denervated samples. **f** Scatter plot to represent the total number of Schwann cells/mm^2^ across denervated samples **g** Nonparametric smoothing linear regression of the total number of SOX10 positive Schwann cells/mm^2^**h** RT-qPCR analysis of SOX10 mRNA expression across denervated samples. **i** Non-parametric smoothing linear regression of the SOX10 RT-qPCR data. Case numbers are attached to each data point for reference to Table 1 with descriptors of whether the samples were collected proximally or distally:* m1* - Proximal part of the denervated stump of the biceps branch of musculocutaneous nerve. *m2* - Distal part of the denervated stump of the biceps branch of musculocutaneous nerve. *b1* - Proximal section of the denervated stump of the brachialis branch of musculocutaneous nerve. *s1* - Denervated stump of suprascapular nerve. *sa1* - Proximal section of the denervated stump of the spinal accessory nerve. *sa2* - Distal section of the denervated stump of the spinal accessory nerve

Immunohistochemistry followed by quantitative analysis of micrographs showed how the number and phenotype of Schwann cells within the denervated samples varied according to denervation time, compared with normal healthy nerve controls. To account for variations in the dimensions of nerves between individuals, cross-sections were quantified in terms of the intra-fascicular density of cells, expressed as immunoreactive cells per mm^2^ cross-sectional area. It is clear from Fig. 3a and b that the total cell density (haematoxylin positive cells) increased after injury to reach a peak after about 90–100 days of denervation. Compared to healthy control nerves, cell density was elevated in samples that were denervated for up to 200 days then this density decreased to lower than healthy controls in the more chronically denervated samples. SOX10 positive Schwann cells represented approximately half of the total number of haematoxylin positive cells in most cases (Fig. 3c and d). The density of SOX10-positive cells also peaked at 90–100 days and then decreased as seen using haematoxylin labelling. In contrast to that seen in injured nerves, the large majority of cells in healthy nerve samples were found to be SOX10 positive.

However, RT-qPCR analysis of SOX10 mRNA expression did not mirror the injury-induced increases in cell density at 90–100 days described above. Instead, SOX10 was upregulated above baseline in all but two of the denervated samples. A trend towards decreasing SOX10 levels was, however, seen after 100 days of denervation (Fig. 3e and f).

**Fig. 4:**
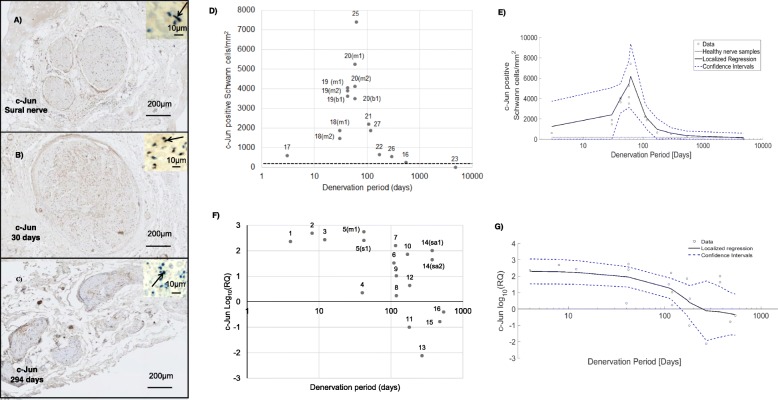
Immunohistochemistry and RT-qPCR analysis of c-Jun in healthy and denervated human nerves. **a**–**c** represent nerve cross sections immunostained for c-Jun (brown) along with haematoxylin and eosin stain. The black arrow in the micrographs indicates a c-Jun positive Schwann cell. The x-axis in **d-g** represents Log (denervation time in days). In **d** and **e** the horizontal black dotted line represents the mean value obtained for the healthy nerve group. **a** Healthy sural nerve. **b** Biceps branch of the musculocutaneous nerve denervated for 30 days. **c** Axillary nerve denervated for 294 days with deteriorated morphology. **d** A scatter plot to represent the total number of c-Jun positive Schwann cells/mm^2^ in denervated samples. **e** Nonparametric smoothing linear regression of the total number of c-Jun positive Schwann cells/mm^2^. **f** RT-qPCR analysis of c-Jun mRNA expression across denervated samples. **g** Nonparametric smoothing linear regression of the c-Jun RT-qPCR data. Case numbers are attached to each data point for reference to Table 1 with descriptors of whether the samples were collected proximally or distally: *m1* - Proximal part of the denervated stump of the biceps branch of musculocutaneous nerve. *m2* - Distal part of the denervated stump of the biceps branch of musculocutaneous nerve. *b1* - Proximal section of the denervated stump of the brachialis branch of musculocutaneous nerve. *s1* - Denervated stump of suprascapular nerve. *sa1* - Proximal section of the denervated stump of the spinal accessory nerve. *sa2* - Distal section of the denervated stump of the spinal accessory nerve

With immunohistochemistry analysis, the number of Schwann cells per mm^2^ showing positive expression of nuclear c-Jun was elevated in the denervated nerves, particularly between 10 and 100 days denervation (Fig. 4a, b, c, d and e). Peak expression was seen at 90–100 days co-incident with the peaks of total cell density and density of SOX10 positive cells. The level of Schwann cell c-Jun expression declined to levels similar to or lower than that of uninjured nerves by around 500 days of denervation (Fig. 4d and e). The RT-qPCR data were comparable, showing that c-Jun expression was increased approximately 140-fold in the samples with the shortest denervation period (4–50 days) and was also increased (to a slightly lesser extent) in most samples up to 200 days (Fig. 4f and g). Beyond this time point c-Jun expression declined, and there was a trend for some of the nerve samples to have c-Jun expression levels lower than the healthy nerve baseline (Fig. 4f and g).

**Fig. 5:**
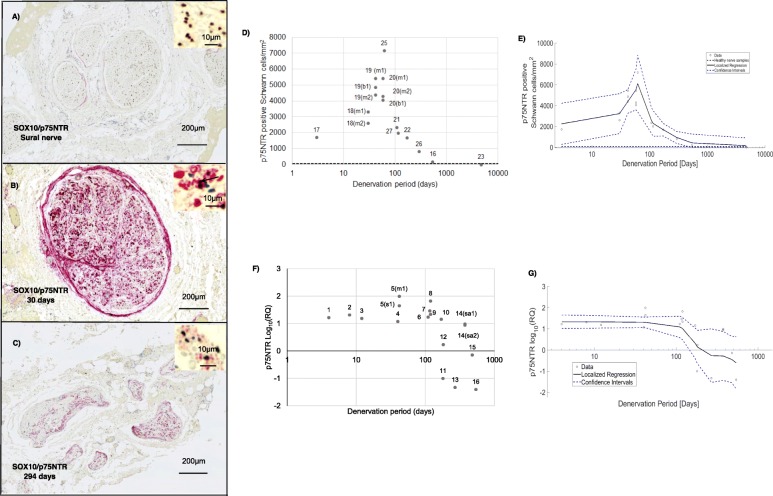
Immunohistochemistry and RT-qPCR analysis of p75NTR in healthy and denervated human nerves. **a**-**c** represent nerve cross sections immunostained for SOX10 (brown) and P75 p75NTR (red) along with haematoxylin and eosin stain. The black arrow in the micrographs indicates a SOX10/p75NTR positive Schwann cell. The x-axis in **d–g** represents Log (denervation time in days). In **d** and **e** the horizontal black dotted line represents the mean value obtained for the healthy nerve group. **a** Healthy sural nerve **b** Biceps branch of the musculocutaneous nerve denervated for 30 days. The brown staining represents a SOX10 positive nucleus whilst the red cytoplasmic staining represents p75NTR positive staining. **c** Axillary nerve denervated for 294 days with deteriorated morphology. **d** A scatter plot to represent the total number of p75NTR positive Schwann cells/mm^2^ in denervated samples. **e** Nonparametric smoothing linear regression of the total number of p75NTR positive Schwann cells/mm^2^. **f** RT-qPCR analysis of p75NTR mRNA expression across denervated samples. **g** Nonparametric smoothing linear regression of the p75NTR RT-qPCR data. Case numbers are attached to each data point for reference to Table 1 with descriptors of whether the samples were collected proximally or distally: *m1* - Proximal part of the denervated stump of the biceps branch of musculocutaneous nerve. *m2* - Distal part of the denervated stump of the biceps branch of musculocutaneous nerve. *b1* - Proximal section of the denervated stump of the brachialis branch of musculocutaneous nerve. *s1* - Denervated stump of suprascapular nerve. *sa1* - Proximal section of the denervated stump of the spinal accessory nerve. *sa2* - Distal section of the denervated stump of the spinal accessory nerve

p75NTR demonstrated a similar trend, with immunohistochemistry analysis showing the number of p75NTR positive Schwann cells per mm^2^ to be elevated particularly between 10 and 100 days of denervation followed by a decline after more than around 80 days of denervation (Fig. 5a, b, c, d and e). The RT-qPCR data were comparable, showing that samples denervated for between 4 and 170 days demonstrated an increase in p75NTR mRNA expression of 10- to 100-fold compared to uninjured nerves. p75NTR expression in most samples that had been denervated for longer declined towards and eventually below baseline (Fig. 5f and g).

Moreover, it was found from the immunohistochemistry and RT-qPCR results that samples collected more distally yielded lower levels of c-Jun and p75NTR than those harvested more proximally (Figs. 4d and f, 5d and f).

**Fig. 6:**
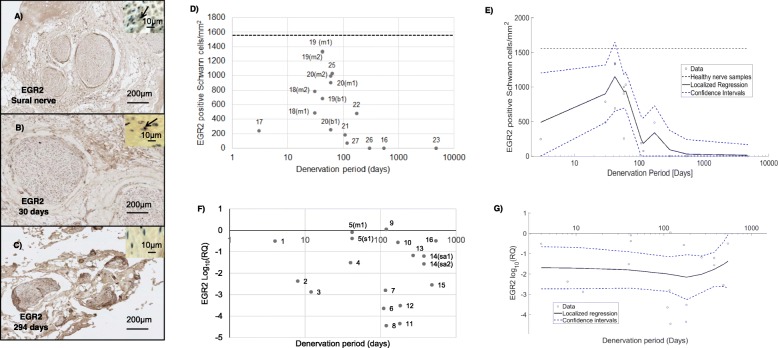
Immunohistochemistry and RT-qPCR analysis of EGR2 in healthy and denervated human nerves. **a**-**c** represent nerve cross sections imunostained for EGR2 (brown) along with haematoxylin and eosin stain. The black arrow in the micrograph indicates a EGR2 positive Schwann cell. The x-axis in **d-g** represents Log (denervation time in days). In **d** and **e** the horizontal black dotted line represents the mean value obtained for the healthy nerve group. **a** Healthy sural nerve. **b** Biceps branch of the musculocutaneous nerve denervated for 30 days. **c** Axillary nerve denervated for 294 days with deteriorated morphology. **d** A scatter plot to represent the total number of EGR2 positive Schwann cells/mm^2^ in denervated samples. **e** Nonparametric smoothing linear regression of the total number of EGR2 positive Schwann cells/mm^2^. **f** RT-qPCR analysis of EGR2 mRNA expression across denervated samples. **g** Nonparametric smoothing linear regression of the p75NTR RT-qPCR data. Case numbers are attached to each data point for reference to Table 1 with descriptors of whether the samples were collected proximally or distally: *m1* - Proximal part of the denervated stump of the biceps branch of musculocutaneous nerve. *m2* - Distal part of the denervated stump of the biceps branch of musculocutaneous nerve. *b1* - Proximal section of the denervated stump of the brachialis branch of musculocutaneous nerve. *s1* - Denervated stump of suprascapular nerve. *sa1* - Proximal section of the denervated stump of the spinal accessory nerve. *sa2* - Distal section of the denervated stump of the spinal accessory nerve

EGR2 expression identified using immunohistochemistry was lower than baseline in nearly all samples, as expected from the involvement of this transcription factor in myelination (Fig. 6a, b, c, d and e). Beyond 1 month of denervation, the proportion of EGR2 positive Schwann cells demonstrated an overall decrease until reaching almost 0 by around 500 days denervation (Fig. 6d and e). RT-qPCR demonstrated a similar trend with most samples demonstrating down-regulation of EGR2 mRNA, by 3000-fold in some cases (Fig. 6f and g).

## Discussion

To help bridge the gap between the extensive studies of rodent Schwann cells in regenerating nerves and their counterparts in injured human nerves, we have examined damaged human nerves for key markers and regulators associated with the rodent Schwann cell injury response. We have paid particular attention to events that take place during chronic denervation of distal nerve stumps, since both in rodents and humans, the deterioration of this tissue during long-term denervation is considered to be an important obstacle to effective nerve repair. For the first time, the markers SOX10, c-Jun, p75NTR and EGR2 have been explored in denervated samples. Moreover, exploration of the markers SOX10, p75NTR and EGR2 in healthy human nerve has been quantified for the first time.

To achieve this objective, this study utilised standardised surgical protocols of human nerve liberation, nerve transfer, autograft and FFMT. Almost 40% of the total samples retrieved from surgery were rejected from the study due to the tissue being unsuitable for RT-qPCR and/or immunohistochemistry analysis. This reflects the significant challenges associated with retrieving human nerve samples from the surgical environment for study in the laboratory [22, 48]. The majority of samples were rejected on the basis of insufficient quantities and quality of RNA. This prompted a recent study to optimise handling of samples in the surgical environment [22].

The data on gene and protein expression obtained from the healthy control nerve population in the present study concurs with the rodent literature, and the axon densities are similar to those reported in studies of human sural nerve samples extracted from cadavers [49, 50]. In addition, there was little variation between samples in the expression of the markers of interest (SOX10, c-Jun, p75NTR and EGR2) between healthy nerve samples from different individuals, assessed using quantitative RT-qPCR and immunohistochemistry analysis suggesting that this was a suitable control group.

Rodent studies have shown that nuclear SOX10 immunoreactivity serves as a specific Schwann cell marker, since in peripheral nerves this transcription factor is selectively and constitutively expressed in Schwann cells [33, 34]. Whilst SOX10 has also been examined in human Schwann cells, this has been largely limited to the study of pathologies such as Schwannoma [36, 51]. The present results show that in healthy human nerve EGR2, c-Jun and p75NTR are expressed in SOX10 positive cells.

Focusing on immunohistochemical detection of axons, it was found that most samples that were denervated beyond 10 days had fewer than 10 axons present. This is consistent with Wallerian degeneration and concurs with neurophysiological reports which have shown that the Compound Muscle Action Potential (CMAP) declines to subnormal and un-recordable levels over 10 days following a nerve injury which has led to the transection of axons [52, 53].

In comparing the density of Schwann cells (SOX10 positive cells) between healthy controls and denervated samples, it was found that nerves that had been denervated for about a month showed approximately 4-fold higher densities of Schwann cells compared to baseline levels. This is consistent with the well documented injury-induced proliferation of Schwann cells in rodent nerves [12–14]. Classically, Schwann cell proliferation has been considered important for regeneration, although this has been called into question by more recent results [54, 55].

In human nerves denervated for longer periods, we observed and quantified another notable aspect of repair Schwann cell biology previously studied in rodents, namely the steady and dramatic reduction of Schwann cell numbers in the denervated stump. Eventually, at 5–7 months, the number of cells fell to levels below even those in uninjured nerves. This decline in cell density during chronic denervation was seen both counting total number of cells positive for haematoxylin and counting SOX10 positive cells only. However, a smaller proportion of the total cell population was SOX10 positive in the chronically denervated samples. This is likely due to an increase in the non-Schwann cell population associated with Wallerian degeneration such as the infiltration of other cells such as macrophages and other immune cells [56–59], which could be investigated in future studies using additional immunohistochemical markers. The gradual loss of Schwann cells from denervated distal stumps is considered to be a major contributor to the creation of an environment that becomes increasingly antagonistic to regeneration over time [11–14, 60]. Contrary to the histological analysis, SOX10 mRNA remained elevated in all but two of the denervated samples. This discrepancy could be attributable to the use of different nerve samples for RT-qPCR and histology as well as alteration of post-translational regulation of SOX10 in the Schwann cells found in injured nerves.

Focusing on markers of Schwann cell phenotype in injured nerves, the expression of all markers was variable between individuals in contrast to that seen in uninjured control nerves, but there were some overall trends. Some of the variability between individuals could be caused by the number of different mechanisms of injury included in the present study (such as avulsions as well as ruptures and lacerations). Age differences between participants (mean age of 34 (±6) years) could also affect the results, since rodent data show that the Schwann cell injury response is subdued in aging animals [61].

From animal models it is known that upregulation of c-Jun is a global amplifier of the reprogramming events that take place in Schwann cells following denervation and are critical for successful nerve regeneration [28, 60, 62]. A key component of this reprogramming is the appearance of a novel set of phenotypes that constitute part of a repair programme, and upregulation of markers that characterise pre-myelinating Schwann cells. p75NTR is one such marker and at the protein level p75NTR has been shown to be regulated by c-Jun. c-Jun and p75NTR are upregulated within hours following injury and continue to increase for a further 7–10 days [63, 64]. During chronic denervation, the expression of these markers in the distal stump then steadily declines. As c-Jun levels decline, functional outcomes become less favourable, while genetic restoration of c-Jun levels in transgenic mice restores regeneration [11, 65–67]. While the role of p75NTR in regeneration is not clear [68–70], the drop in p75NTR expression during chronic denervation has been used as a marker of repair Schwann cell deterioration [63, 64].

For the first time, this study has shown that a similar pattern of regulation is seen in human nerve regeneration. c-Jun and p75NTR increase in acutely damaged samples (within the first month) and decline in chronically denervated samples. This finding provides new information that can inform the clinical management of nerve-injured patients. Clinical studies suggest that optimal muscle reinnervation is dependent upon a sufficient quantity and quality of motor units being established at the target organ within 1-year following injury [20, 71]. This time-frame has been largely based on the understanding of degenerative changes at the motor endplate and within the denervated muscle [18–20, 72, 73]. Importantly, the present study provides new evidence suggesting that the repair phenotype of Schwann cells also fades over a shorter time period of around 100 days following injury, resulting in an environment increasingly less supportive of regeneration. Moreover, the results show that in human Schwann cells**,** c-Jun and p75NTR expression are associated, as previously seen in rodents**,** suggesting that the basic molecular features which underpin nerve regeneration in humans and rodents are comparable.

The changes in the mRNA reported here may also mirror changes at the level of the spinal cord. Animal models have shown that shortly after neural trauma, injury-induced excitation signals are transduced retrogradely from neuronal and non-neuronal cells to their own injured cell body [74]. An array of molecular responses have been identified leading to the dysregulation of neurotrophic factors, neurotrophic receptors, neuropeptides and transcription factors [74]. Specifically, the upregulation of c-Jun and p75NTR and down-regulation of EGR2 represent key changes in the creation of a neuronal phenotype that is supportive of regeneration, which fades over time leading to the reduced regenerative capacity of chronically axotomised axons [74].

The nonparametric linear regression analysis of Schwann cell numbers and c-Jun, p75NTR and EGR2 expression during chronic denervation summarises key temporal changes in repair Schwann cells after injury. With further data this could become a useful tool in assessing and predicting changes associated with denervation in PNI patients. It is notable that the magnitude of the expression fold changes in gene expression between control and damaged human nerves, presented in the RT-qPCR data, are significantly larger than those reported in rodent studies [28, 75–77]. To accommodate this range, the present data are displayed as Log_10_ (RQ) changes, rather than Log_2_(RQ) changes which are reported in a number of rodent studies [28, 75–77].

Where a sample had been collected and dissected into proximal and distal sections from the same nerve (case [14, 18, 19 and 20]), samples which were collected more distally expressed lower quantities of repair Schwann cell markers (c-Jun and p75NTR) than their more proximal counterparts. This observation, whilst based on a limited number of samples, suggests that Schwann cell phenotype becomes less supportive of regeneration in more distal nerve segments, an observation that warrants further exploration.

Down-regulation of the molecular pathways associated with myelination is another key component of the repair programme that is governed by c-Jun in rodents [30, 60, 78, 79]. EGR2 is a marker of myelination and its absence or malfunction has been linked with a number of myelinopathies [29, 31]. The expression of EGR2 was down-regulated in all denervated samples presented in this study, indicating reversal of myelin differentiation. This suggests that after injury the molecular machinery of myelination in human Schwann cells is regulated in a similar way to that in rodents**.**

This study should be interpreted in light of its limitations. Although samples were assumed to be denervated based on intra-operative neurophysiology, a few samples demonstrated some positive neurofilament staining. This is perhaps attributable to neurofilament positive autonomic/afferent fibres. Such axons would not have been detected in the functional screening and would therefore not have been excluded from the study. To a small degree, the presence of these axons may have influenced the local cellular environment, meaning that not all of the Schwann cells will have shifted towards a repair phenotype. In addition, only a small number of healthy nerve samples were used for comparison with denervated samples (three samples for RT-qPCR and two samples for histology). This limitation reflects the ethical and practical challenges associated with obtaining healthy nerve samples. Even when it is possible to retrieve small samples of healthy nerve during surgical procedures, the yield of RNA is often reduced to levels sub-optimal for quantification [22]. In addition, the small size and morphology of these samples can make them inappropriate for histological analysis when complete transverse sections cannot be obtained. A further limitation was that this study only obtained a small number of acutely denervated samples (three samples retrieved less than 30 days following the initial injury). In the general trauma population, nerve injuries often occur secondary to severe vehicular collisions [80, 81] which is reflected in the present study, accounting for 18 out of 27 of injuries (67%). As a result, many patients present with co-morbidities that require treatment before investigation of a suspected nerve injury, leading to a substantial delay between the initial injury and reconstructive nerve surgery where the samples can be retrieved. In addition, patients are often observed for 3 to 6 months for spontaneous functional recovery following blunt trauma before surgical repair is considered [82]. For these reasons, the retrieval of acutely denervated (less than 10 days between injury and surgery) samples was challenging. This study would also benefit from the inclusion of more samples at standardised time points. However, this is challenging due to the heterogeneous nature of PNI, the rarity of the injuries as well as the practical and ethical challenges associated with harvesting human nerve tissue for study in the laboratory [21, 22]. Lastly, only one sample from a female was retrieved in the present study (case number 4). This sample was found to have lower quantities of the c-Jun and p75NTR mRNA compared to males at a similar denervation time period (Figs. 4f and 5f). This suggests that the repair Schwann cells in this sample are less supportive to neuronal regeneration. This contradicts evidence from rodent models that suggests females exhibit a faster rate of neuronal regeneration compared to males [83, 84]. It has been shown that this differential may be attributable to a repair Schwann cell phenotype in the distal stump that is sustained for a longer period of time following injury in females compared to males [83, 84]. This warrants studies of additional female nerve samples to compare the time course of repair Schwann cell deterioration to the findings presented here.

## Conclusion

In summary, this study provides new insights into some of the key cellular and molecular features that underpin the regenerative capacity of the human PNS, providing additional explanations for clinical observations and reports. It was found, first, that two major genes associated with repair Schwann cells in rodents, c-Jun and p75NTR, are also up-regulated in acutely injured human nerves, an observation that encourages the view that rodent models are relevant for learning about human nerve injury. Second, as in rodents, the expression of both of these genes declines during long-term denervation. In rodents, diminishing c-Jun and p75NTR levels mark the general deterioration of repair cells during chronic denervation, a process thought to be a major obstacle to effective nerve repair. The down-regulation of c-Jun and p75NTR reported here provides the first molecular evidence that also in humans, repair cells deteriorate during chronic denervation, and provides markers with which this critical process can be monitored.

## Supplementary information

**Additional file 1.** Methods and materials Additional explanation of statistical analysis implemented, positive controls used for immunohistochemistry staining and further data from uninjured nerve samples.

## Data Availability

The datasets used and/or analysed during the current study are available from the corresponding author on reasonable request.

## Abbreviations

PNI: Peripheral Nerve Injury
PNS: Peripheral Nervous System
CNAP: Compound Nerve Action Potential
FFMT: Free Functioning Muscle Transfer
p75NTR: Neurotrophin Receptor
RT-qPCR: Real Time-quantitative Polymerase Chain Reaction
REC: Research Ethics Committee
DAB - 3,3′: Diaminobenzidine
HRP: Horseradish peroxidase
HIER: Heat-induced epitope retrieval
RT: Reverse transcription
cDNA: Complementary DNA
GOI: Gene of interest
HKG: Housekeeping gene
NTC: Negative Controls
C_T_: Threshold cycle
C_T:e_: C_T_ value for endogenous control
C_T:c_: C_T_ value for calibrator
RQ: Relative quantification
CMAP: Compound Muscle Action Potential
