# Supplementary material for: Characterising cellular and molecular features of human peripheral nerve degeneration
Source: Acta Neuropathol Commun. 2020 Apr 17;8:51. doi: 10.1186/s40478-020-00921-w (PMC7164159; doi:10.1186/s40478-020-00921-w)
Supplement: Supplementary file 1 — Additional file 1. Methods and materials Additional explanation of statistical analysis implemented, positive controls used for immunohistochemistry staining and further data from uninjured nerve samples. [file 40478_2020_921_MOESM1_ESM.pdf]

Additional file 1

Statistical analysis

The aim of this aspect of the work was to understand the trends in the data available. Trends are commonly uncovered by fitting polynomials of different orders to data. However, for small data sets this may result in an over fit, not least as the variance in the data is not accounted for. Here, therefore, nonparametric smoothing linear regression was implemented (1). This method groups data points into bins and then performs a linear regression to the points in each bin. The size of the bins (termed bandwidth) is a key parameter choice to ensure the data are well represented without biases. To this effect, a cross-validation method was implemented: the data were randomly sampled into two sets; a range of bandwidth values were tested over several randomly sampled sets of the data. The bandwidth was chosen as that which attained the smallest least mean square error on average for all the prediction data sets. After identifying the optimal bandwidth, the robustness of the model was tested. This was achieved through a bootstrap methodology, which involved generating thousands of random data samples with the same size as the source data, and applying the linear smoothing algorithm with the bandwidth established over all these samples. From this a mean and standard deviation of all the resulting curves was calculated. Through this method, data trends were more clearly illustrated to facilitate discussion and interpretation.

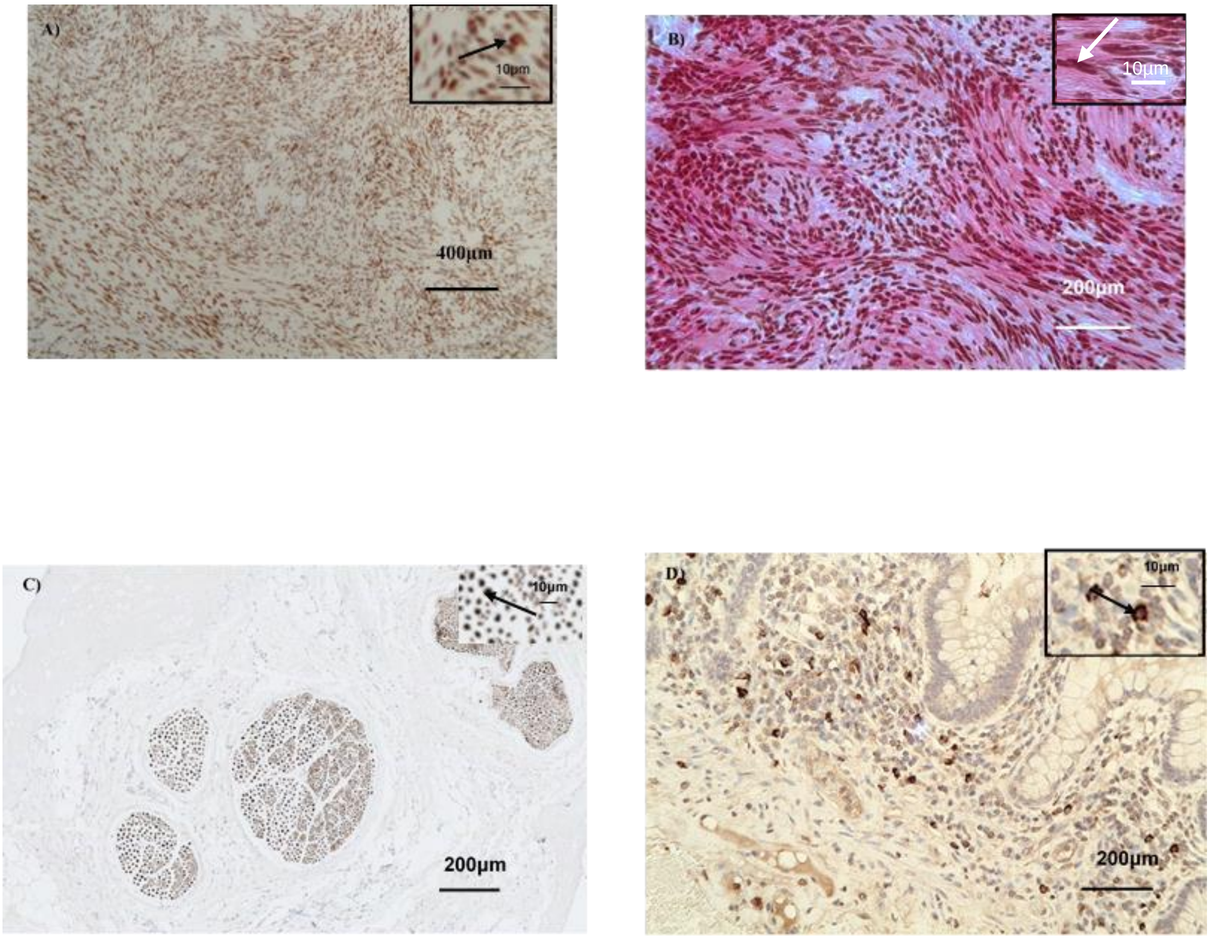

Fig. 1 – Positive controls for immunohistochemistry.

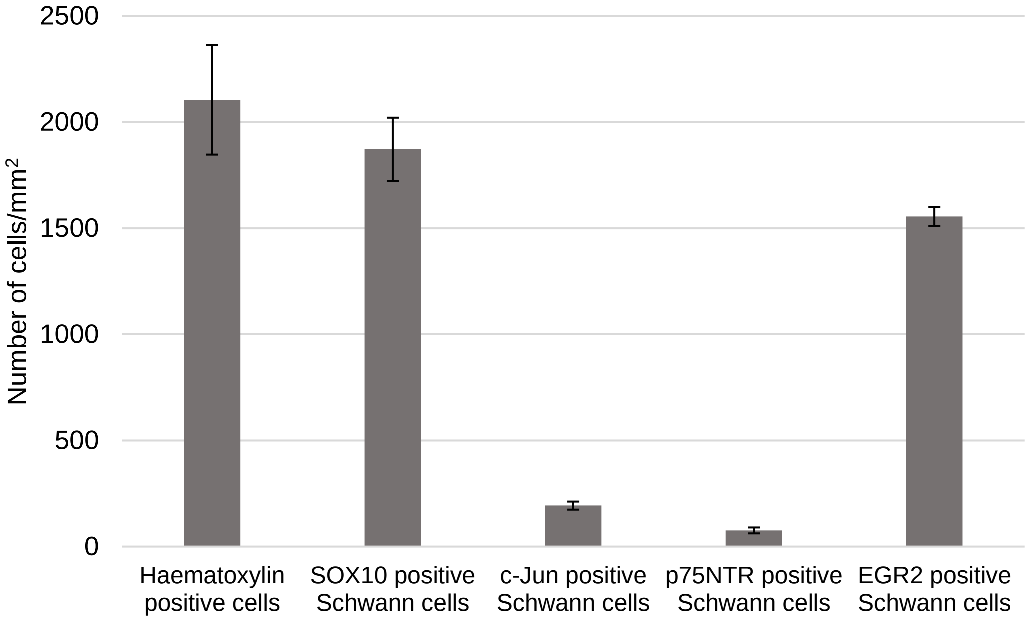

Fig. 2 – Quantitative immunohistochemistry for healthy nerves.

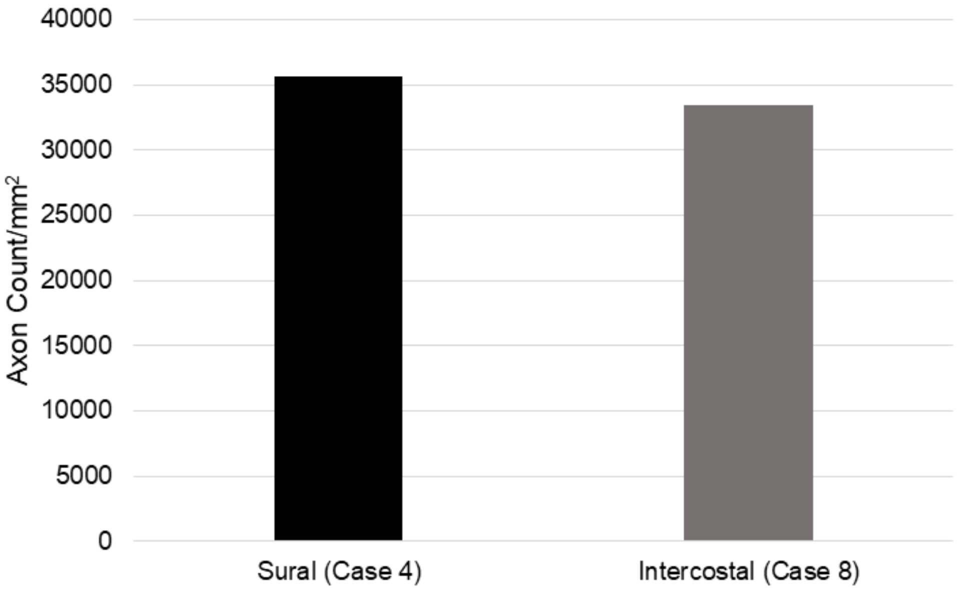

Fig. 3 – **Axon counts in healthy nerves.**

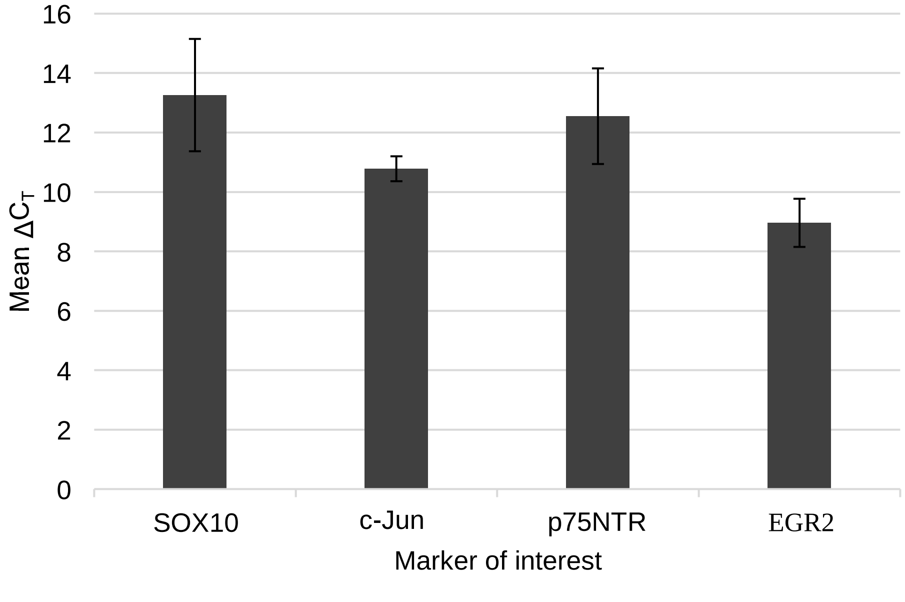

Fig. 4 – **Real Time-quantitative Polymerase Chain Reaction data for healthy nerve group.**

Additional file 1 figure legends

Fig. 1 – **Positive controls for immunohistochemistry**. The black arrows in the micrographs indicate positive staining. **A)** Staining of Schwannoma for c-Jun (brown), **B)** co-staining of Schwannoma tissue for SOX10 (brown) and p75NTR (red), **C)** neurofilament (brown) staining of uninjured sural nerve, **D)** human colon stained for EGR2 (brown). The black arrows indicate cells that are positive for the marker of interest.

Fig. 2 – **Quantitative immunohistochemistry analysis of healthy nerves.** Bar charts to represent quantification of immunohistochemically stained healthy nerve samples (case number 4 (sural) and case number 8 (intercostal) as reported in ***Table 1***) for the markers SOX10, c-Jun, p75NTR and EGR2.

Fig. 3 – **Axon counts in healthy nerves.** A bar chart to represent axons/mm^2 in the healthy sural (Case Number 4) and intercostal nerve samples (case number 8) as reported in ***Table 1***.

Fig. 4 – **RT-qPCR data for healthy nerve group.** Bar chart to represent the mean $\Delta C_T \pm 1$ Standard Deviation (SD) of the genes SOX10, c-Jun, p75NTR and EGR2 across the healthy nerve population (from case numbers 4 (sural nerve), 8 (intercostal nerve) and 24 (intercostal nerve) as reported in ***Table 1***).

References

1. Härdle W, Schimek M. Statistical Theory and Computational Aspects of Smoothing: Proceedings of the COMPSTAT'94 Satellite Meeting Held in Semmering, Austria, 27–28 August 1994: Springer Science & Business Media; 2013.
